# Antidiabetics and antihypertensive medications use in Morocco: A pharmacoepidemiological descriptive study

**DOI:** 10.4102/phcfm.v12i1.2195

**Published:** 2020-06-15

**Authors:** Elyamani Rida, Abdelmajid Soulaymani, Zineb Serhier, Hinde Hami, Mokhtari Abdelrhani

**Affiliations:** 1 Laboratory of Genetics and Biometry, Department of Biology, Faculty of Sciences, Ibn Tofail University, Kenitra, Morocco; 2 Laboratory of Medical Informatics, Department of Social Medicine and Community Health, Faculty of Medicine and Pharmacy, University of Hassan, Casablanca, Morocco

**Keywords:** pharmacoepidemiology, antidiabetics, antihypertensive, Morocco, pharmacovigilance, medication safety

## Abstract

**Background:**

In Morocco, and many other African countries, there is a paucity of antihypertensive and antidiabetics use amongst the general population.

**Aim:**

To investigate the epidemiological profile of antihypertensive and antidiabetics use and analysis their adverse reactions.

**Setting:**

This study was conducted in the prefecture of Figuig, Morocco.

**Methods:**

A cross-sectional descriptive study. Data was collected using semi-structured questionnaire about their pharmacological treatment and presented using descriptive statistical analysis.

**Results:**

Sample of 244 subjects, predominantly women 58.15% (*p* < 0.03) composed of diabetic patients 56.96% (*n* = 139) and hypertensive patients 43.03% (*n* = 105). After adjustments, 60.24% of all patients were under monotherapy. The diabetics were being treated using the Biguanide class (26.92%), insulin (20.0%) and sulfonylureas (10.0%) while hypertensive patients were treated by Calcium Channel Blockers (27.36%), Angiotensin Converting Enzyme Inhibitors (21.05%), Angiotensin T-Blockers (16.84%), Diuretics (7.36%) and β-adrenergic receptors blockers (3.15%). In total, 23.00% of all subjects have experienced negative side-effects, mostly, reported (90.38%) to health professionals and 23.52% of them have interrupted temporarily or try to change their treatment. Gastro-intestinal problems were the most adverse reactions reported (11.11%) followed by headache, dizziness and tinnitus (6.66%) and asthenia, feeling sick and feeling of faintness (5.33%).

**Conclusion:**

Managing diabetes and hypertension entails a lot of public challenges and requires more focus and interest, especially amongst the illiterate population in remote areas. Some of the suggested ways to help face the problem include the introduction of new innovative measures, systems of fellow-up and adverse reactions.

## Introduction

Diabetes and hypertension remain the most common public health problems in the world and are the major risk factors for cardiovascular diseases that are the leading cause of death.^1^ Globally, both diseases affected nearly 2 billion people, with 1.4 billion hypertensive subjects and 425 million diabetic subjects, and accounted directly for 7.5 million and 1.6 million deaths respectively in 2017 (World Health Organization). The pharmacological treatment using antihypertensive and antidiabetics is core to any medical procedure for resolving these chronic diseases in any population, especially in developing countries where the availability of the pharmaceutical products remains a real challenge for governments in terms of both the technological innovation (generics penetration) and the economic burden.^2^ Daily use of these medications, and for long terms, is a complicated process affected by the psychological adjustment of subjects, their socioeconomic status and the mixture of compounds for treating the chronic conditions, regarding their effectiveness, their adverse reactions, their cost and their availability. To achieve positive therapeutic goals represented by positive clinical outcomes, and therefore prevent macrovascular and microvascular complications, subjects have to adhere to long-term guidelines and strategies for diets and pharmacological treatment following prescribed daily doses.^3,4^ Generally, for diabetes and hypertension, there are two major indications for changing the medication: a therapeutic failure and/or the appearance of serious adverse reactions or discomfort. The antidiabetics have varying effectiveness and with several side effects,^5^ whilst the hypertension treatment has been revolutionised by the introduction of many new classes; particularly, the calcium channel blockers (CCB), the angiotensin converting enzyme inhibitors (ACEI), the angiotensin receptor blockers also called (also called T-blockers), the diuretics and other drugs that are cost-effective safe. In Morocco, as in many other African countries, there is a paucity of use of antihypertensives and antidiabetics amongst the general population. This study aimed to contribute to developing a picture of the pharmacoepidemiological profile of antihypertensives and antidiabetics in Morocco for academic purposes.

## Methods

### Location

This study was conducted in the province of Figuig in south-eastern Morocco. This province has a semi-arid climate with an area of 55 990 km^2^, which represents 7.92%^6^ of the Moroccan land mass, and is suitable for the agricultural livestock.^7^ According to the national census of the Moroccan population (2014), the total population of the province included 138 325 inhabitants defining a density of 2.5 inhabitants/km^2^ and 50% of the population living in rural areas.^8^ The global multi-dimensional poverty is at 13.1% whilst the rates of illiteracy and health privation were 52.8% and 9.6% respectively (2014).^9^ The official number of diabetic subjects recorded in the prefecture was 1316 subjects (788 women vs. 528 men) and 1076 hypertensive subjects (699 women vs. 377 men) (2016).

### Study design

This is a cross-sectional study funded by the Ministry of Health through a partnership with Ibn Tofail University. We followed the annual programme of medical caravans and medical visits to investigate diabetic and hypertensive subjects across different geographic locations of the prefecture. Patients aged 30 years and more who came for consultations were selected randomly and were screened using a semi-structured questionnaire, and their anthropometric and biological parameters were measured. Patients with advanced symptoms were transferred to specialised consultations, and those with medical prescriptions were directed to the pharmacy to be treated. Pregnant women, recently diagnosed patients (less than 1 year) and subjects not able to communicate were not included in this work.

### Statistical analysis

Data were transferred from the questionnaire paper into an electronic database. We performed the analysis using Epi-Info software, and after adjustment we used univariate descriptive statistics to express the characteristics of patients. Our results were expressed as mean (±), standard deviation (s.d.), percentage (%) or as number of cases (*n*). We used the *t*-test to compare means and the *χ*^2^-test to compare proportion.

### Ethical consideration

The study received ethical clearance from Ibn Tofail University and Ministry of Health of Morocco on 01 December 2016 (ethical clearance number: P1-12/16-LGB-MH). A contract and partnership were formed between Ibn Tofail University and the Ministry of Health to support and authorise this study following the ethical standards. All procedures in this study were in accordance with the ethical standards of the National Committee and the 1964 Helsinki Declarations. Participants were asked to share their information for scientific research purposes; those who refused were screened for their medication and their data were not included. Participants were assured regarding the confidentiality of their information. The special terminologies and words have been accepted to express medical meanings to ensure the effectiveness of the communication, because most subjects were not well-versed with the local language. Considering the local traditions, wives were interviewed and screened in the presence of their husbands.

## Results

Our sample comprises 244 subjects with a mean age of 60.64 (s.d. ± 12.90) years which form a population of diabetic subjects (56.96%; *n* = 139) and hypertensive subjects (43.03%; *n* = 105) and were predominantly women (58.15%; *n* = 142) (*p* < 0.03) selected randomly from various geographic locations, rural areas (59.01%) and urban areas (40.98%) in one prefecture. Table 1 summarises the main characteristics of the population.

**TABLE 1:** Characteristics of the population.

Characteristics	Diabetics		Hypertensive		Total	
	%	*n*	%	*n*	%	*n*
Number	56.96	139	43.09	105	100	244
Mean age (years)	57.16±12.47	-	65.26±12.04		60.64±12.90	
Men	23.77	58	18.03	44	41.80	102
Women	33.19	81	25.0	61	58.19	142
Disease duration	7.3±5.5		6.33±4.45		6.82±5.02	
Education	17.62	43	6.55	16	24.18	59
Medical insurance	92.08		93.33		92.62	
Other chronic condition (*epilepsy, asthma, rheumatism, etc.*)	5.75	-	8.57		6.96	
No treatment	3.68	9	4.09	10	7.78	19

### Pharmacotherapy antihypertensive and antidiabetics

**FIGURE 1:**
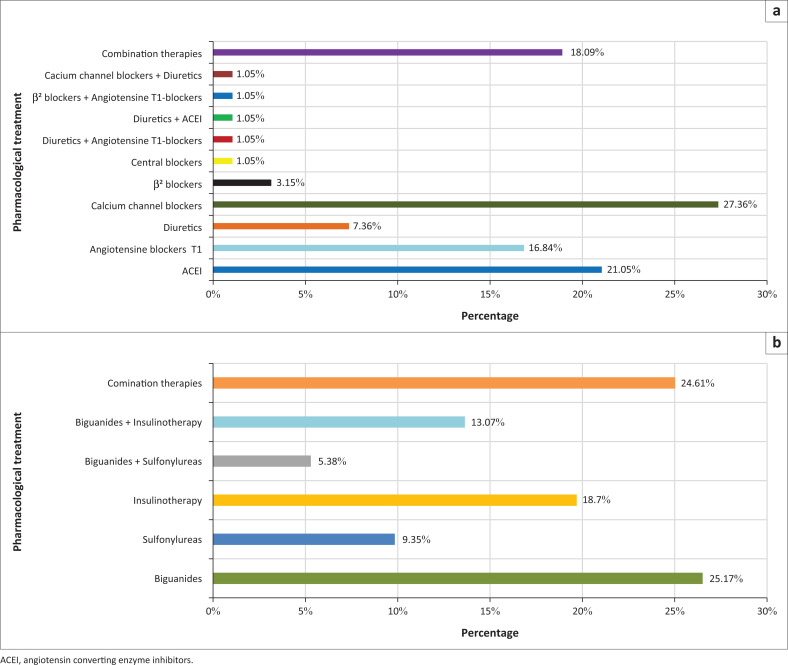
Distribution of therapies using antihypertensive and antidiabetics over patients (a) Pharmacological treatment of hypertensive subjects; (b) Pharmacological treatment of diabetic subjects.

Most subjects had a medical insurance (92.62%) and low educational level (24.18%). Only 7.78% of all subjects were not following any pharmacological treatment for their chronic conditions. The distribution of pharmacological treatments over patients is shown in Figure 1.

Regarding their therapeutic conception and after adjustments, 60.24% of all patients were undergoing monotherapy (hypertension 29.91% (*n* = 73) and diabetes 30.32% (*n* = 74)) and the rest used combinations therapies for single or multiple chronic conditions. Several compounds from various pharmacological classes were employed to treat these conditions. Thus, for diabetes, the main compounds used in monotherapy are the biguanides (26.92%), insulin therapy (20.0%) and sulfonylureas (10.0%). Patients under bi-therapies (18.46%) were divided into two major associations: biguanides with insulin (13.07%) and biguanides with sulfonylureas (5.38%). The rest (24.61%) were under combinations of antidiabetics, antihypertensive medication, because they were affected simultaneously by diabetes and hypertension, with cholesterol lowering (statins) or with antithrombotic.

Hypertensive subjects use CCB (27.36%), ACEI (21.05%), angiotensin T-blockers (16.84%), diuretics (7.36%) and b-adrenergic receptors blockers (β-blockers) (3.15%) as part of the monotherapy treatment. The bi-therapy antihypertensive (4.21%) uses combinations of diuretics with ACEI, β-blockers with T-blockers, CCB with diuretics and diuretics with T-blockers. The rest (20.03%) uses combinations of antihypertensive, cholesterol lowering (statins), antithrombotic and antiarrhythmic drugs. The general prevalence of polypharmacy (drugs ≥ 5) is 1.22%; all of them were diabetics (2.15%) using combination therapies of antidiabetics, antihypertensives, cholesterol lowering drugs and antithrombotics.

Combination therapies include various therapies, such as antidiabetics, antihypertensive, cholesterol lowering, antithrombotic and antiarrhythmic drugs.

### Pharmacovigilance

Almost 23.00% of all subjects have experienced negative side effects; this is distributed into 12.43% of diabetics and 10.66% of hypertensive patients. These adverse reactions were reported (90.38%) to health professionals and 23.52% of them had their treatments interrupted temporarily or changed. Interestingly, men report the side effects to health professionals more than women (100% vs. 86.11%) and interrupt the treatment or try to change their treatment more compared to women (33.33% vs. 19.44%) (*p* < 0.02).

Gastrointestinal problems were the most adverse reactions reported by 11.11% of subjects (11.57% in the hypertension group and 10.57% in the diabetes group). The second most reported side effects include headache, dizziness and tinnitus (6.66%) which are observed more frequently amongst the hypertension group than the diabetes group (10.52% vs. 3.84%). Side effects such as asthenia, feeling sick and a feeling of faintness were reported with a frequency of 5.33%. Table 2 describes the main side effects reported in the survey.

**TABLE 2:** Description of side effects reported in the survey.

Side-effects reporting frequencies	Hypertension group		Diabetes group		Total sample	
	%	*n*	%	*n*	%	*n*
Bradycardia	1.05	1	0%	-	0.44	1
Stomach ache, nausea, vomiting, abdominal discomfort	11.57	11	10.76	14	11.11	25
Dizziness, headache, tinnitus	10.52	10	3.84	5	6.66	15
Asthenia, feeling sick, feeling of faintness	5.26	5	5.38	7	5.33	12
Coughing	1.05	1	0	-	0.44	1
Delivery product’s injection point	0	-	3.07	4	1.77	4

## Discussion

This is the first observational pharmacoepidemiological investigation of antidiabetics and antihypertensives utilisation in one prefecture in Morocco.

In Morocco, since 2011, the generalisation of the basic healthcare insurance, especially amongst needy populations, has been improving the healthcare access, chiefly the pharmacological treatment.^10^ Affected by the expansion of the national pharmaceutical market, the consumption of these drugs has increased drastically in the last decades, encouraged by the generic penetration and advancement of the therapeutic innovations in Morocco.

In terms of antidiabetics, our findings pointed out the widespread use of the biguanides, both in monotherapy and in association with various antidiabetic agents, and defined the same epidemiological pattern in other developing African countries.^11,12,13^ The national pharmaceutical market of antidiabetics progressed from ranging over six specialties (1991) to 16 specialties (2005) translating the huge demands for these agents, evolving from 1.9 to 14 DDD/1000 inhabitants/day (DDD = defined daily dose) from 1997 to 2014.^14^ Analyses show that the national consumption of sulfonylureas and metformin have increased by four and 10 times respectively from 1997 to 2004. Our results show that 21.37% of diabetic patients experienced discomfort with their pharmacological treatment. A prospective study^15^ of oral antidiabetics amongst Moroccan diabetic patients indicated that 50% of those patients experienced adverse reactions, mostly related to metformin (95%) and sometimes did require the interruption of treatment, whilst sulfonylureas were mainly linked to hypoglycaemia as their side effect (0.5%).

Most of the antidiabetics described in our survey are available in public health facilities as part of the national health policy for managing chronic diseases (2012).^10^ The class of biguanides is generally well-tolerated compared to insulin, is inexpensive, acts by decreasing the insulin resistance, inhibits hepatic gluconeogenesis and opposes the action of glucagon. Their preventive actions on the cardiovascular system were confirmed in 53 observational and experimental studies^16^ (Hazard ration HR 0.78, 95%, CI 0.73–0.83, *p* < 0.00001) as compared to the insulin group. Their main adverse reactions include nausea, abdominal discomfort and some diarrhoea.^17^ Generally, metformin has to be taken with meals in an increasing dose as tolerated. A retrospective cohort study^16^ conducted in England and Wales has linked insulin with an increased risk of composite non-fatal acute myocardial infraction, which causes death and non-fatal stroke (HR 2.6, 95%, CI 1.9–3.4). The association of biguanides with insulin^16^ was linked to gastroduodenal discomfort, weight loss and hypoglycaemia. The class of sulfonylureas^17^ acts as insulin secretagogues, and as β-cell pancreatic dysfunction progresses, they become less effective. They are inexpensive, but their main adverse reaction includes symptomatic episodes of hypoglycaemia.

In Morocco, the use of antihypertensive drugs increased from 0.08 to 10.65 DDD/1000 inhabitants/day between 1991 and 2010 with a dominance of CCB drugs (82.09%) followed by ACEI drugs (48.29%).^18^

Our findings reveal that drugs acting on the renin–angiotensin aldosterone system (RAAS) are widely used in monotherapy (37.89%) represented by two major pharmacological classes: ACEI and T-blockers. Generally, they are considered as the first line and are quite effective in the management of hypertension.^19^ The most important adverse reaction^20^ in this group is represented by dry cough because of increase in bradykinin concentration which may constitute an indication to switch from ACEI to T-blockers. Other side effects include angioedema, hyperkalaemia and diarrhoea.^21^ When combined with β-blockers, they have additive cardio-protective actions with less antihypertensive action.^22^ The second major pharmacological class was represented by CCB represented with its two subgroups: the Phenylalkylamine (verapamil) and dihydropyridine (amlodipine).^23^ Their pharmacological antihypertensive action resulting in vasodilatation is considered more effective than the ACEI and β-blockers, especially in the African population.^24^ Their adverse reactions are dose dependent, because of their vasodilatation actions, and essentially include gastrointestinal problems (constipation associated with Verapamil in 13%), headache, dizziness, light-headedness, flushing, hypotension and peripheral oedema (10% – 20% of all patients) more commonly found in women and probably because of an increased capillary pressure and for which the association with diuretics may not reveal this oedema, whilst the association with ACEI or T-blockers may reduce or prevent it.^23,25^ The results of 22 clinical trials^24^ show lower incidence of diabetes compared to diuretics and β-blockers but greater than ACEI and T-blockers. Their association with diuretics is acceptable considering that they excrete sodium preventing the volume depletion that occurs with diuretics and their help in reduction of cardiovascular risks.^26^ The third antihypertensive class represented by the diuretics contains three major groups of compounds depending on their pharmacological actions: the thiazides (hydrochlorothiazide and indapamide), the loop diuretics (furosemide) and the potassium spearing diuretics (spironolactone).^27^ The main adverse reactions of diuretics include headaches, dizziness, increased blood glucose, gout, diarrhoea, kidney failure and irregular heartbeat. They reduce the intravascular volume and activate the Renin Angiotensin Aldosterone system (RAAS) leading to vasoconstriction and the retention of sodium and water.^28^ Therefore, the association with an ACEI or T-blockers attenuates this irregularity and ameliorates the hypokalaemia with an increasing risk of hyperkalaemia.^29^

The β-blockers are used as a second line of hypertension management, especially amongst patients who survived an acute myocardial infraction, heart failure and atrial fibrillation by reducing the heart’s rhythm. When compared to ACEI, T-blockers and CCB, the β-blockers have a reduced action on hypertension.^30,31^ Their adverse reactions, which can be explained by understanding their molecular mechanism and competitive antagonist action on c-adrenoceptors, include interference with heart rate, hypotension, hypoglycaemia, abdominal pain, nausea, vomiting, constipation, dizziness, depression and many others.^32^

In this survey, 6.9% of all participants have associated chronic conditions, such as asthma, rheumatism, psychological disorder and epilepsy which increase the complexity therapeutic not only for the subjects but also for health professionals as it increases the risk of drug–drug interactions. Our findings show an adjusted prevalence of polypharmacy^33^ in 1.22% observed diabetic subjects. We consider two major factors that determine the success of combination therapies:

1. Tolerability: Usually, antidiabetic and antihypertensive drugs have pharmacological actions and adverse reactions that are dose dependent, hence, low-dose initiations are always preferable.
2. Adherence: The subject’s ability to respect the therapeutic guidelines, particularly, with multiple drugs that have to be taken at the same time and/or in increasing number of doses per day and amongst illiterate subjects which may affect patients’ compliance negatively, who will try to change or interrupt its treatment. We found that 23.53% of subjects who did experience adverse reactions have interrupted temporarily or tried to change it by themselves without any medical consultations which exposed them to negative clinical outcomes and complications. Morocco has an advanced system of pharmacovigilance compared with other Arabic countries and full membership with the World Health Organization (WHO) Collaborating Center for International Drug Monitoring (WHO-Uppsala Monitoring Center). The system has a centric structure based on the National Center of Pharmacovigilance (launched in 1989) and the National Pharmacovigilance Committee.^34^ Spontaneous reports of adverse reactions are made by health professionals essentially depending on their initiative and motivation. In our investigation, the vast majority of adverse reactions were reported to health professionals who probably were not sufficiently trained to deal with these reactions or take them seriously.

## Limitation and perspectives

Our sample was representative of registered patients in the prefecture (nearly 10%); extrapolation to the general population should consider the ethnic diversity (Arabs and Amazigh) and the socioeconomic variations that characterise the Moroccan population and which may affect the lifestyles.

Further investigations are required to define new strategies of side effects of medication in long-term use.

## Conclusion

Managing diabetes and hypertension still pose a lot of public health challenges and requires more focus and interest, especially amongst the illiterate population in remote areas. The management measure should include the introduction of new innovative measures, systems of follow-up and adverse reactions management, at the individual, regional and national levels.
